# Supplementary material for: Cardiorespiratory physiological perturbations after acute smoke-induced lung injury and during extracorporeal membrane oxygenation support in sheep
Source: F1000Res. 2020 Jul 24;9:769. [Version 1] doi: 10.12688/f1000research.24927.1 (PMC7481850; doi:10.12688/f1000research.24927.1)
Supplement: Peer review history

# Peer review: Reviewer response for version 1

**DOI:** 10.5256/f1000research.27501.r69959

**Published:** 2020-09-09

**Authors:** Noah H Hillman

**Affiliations:** 1 Division of Neonatology, Cardinal Glennon Children's Hospital, Saint Louis University, Saint Louis, MO, USA

The manuscript provides information on the physiology from ECMO experiments in adult sheep. The article discussed everything from the physiology of the lung disease and the parameters used for the ECMO system. It gives some guidance for other researchers working on sheep models of ECMO or lung injury responses.

 The information on the sedation drugs used will give guidance to other utilizing these therapeutic surgical options. Although mentioning what a research team used in an experiment is not entirely the same as proving these are the optimal treatment for the animals.

 The conclusions in abstract are very broad, as it is unclear how a study on smoke inhalation helps with management of tick paralysis unless these animals get severe lung disease.

Is the work clearly and accurately presented and does it cite the current literature?

Yes

If applicable, is the statistical analysis and its interpretation appropriate?

Yes

Are all the source data underlying the results available to ensure full reproducibility?

Yes

Is the study design appropriate and is the work technically sound?

Yes

Are the conclusions drawn adequately supported by the results?

Yes

Are sufficient details of methods and analysis provided to allow replication by others?

Yes

Reviewer Expertise:

Neonatal lung disease and sheep ventilator research

I confirm that I have read this submission and believe that I have an appropriate level of expertise to confirm that it is of an acceptable scientific standard.

# Peer review: Reviewer response for version 1

**DOI:** 10.5256/f1000research.27501.r70256

**Published:** 2020-09-03

**Authors:** Gabrielle C Musk

**Affiliations:** 1 Animal Care Services, University of Western Australia, Nedlands, Australia

This manuscript provides extensive physiological data collected opportunistically during sheep studies of acute lung injury. The manuscript is well written and comprehensive. I have a few minor comments to address:

- Include details on the fate of the animals at the end of the experiment.
- Include brief details of the anaesthesia protocol in the materials and methods instead of just the reference to previous work.
- Final paragraph page 13: suggest using the term nociception instead of pain as the animals were unconscious. Also tachycardia can occur with ketamine administration so may have contributed.
- Final paragraph page 20: rephrase 'already out there' to 'previous published' or similar.

Is the work clearly and accurately presented and does it cite the current literature?

Yes

If applicable, is the statistical analysis and its interpretation appropriate?

Yes

Are all the source data underlying the results available to ensure full reproducibility?

Yes

Is the study design appropriate and is the work technically sound?

Yes

Are the conclusions drawn adequately supported by the results?

Yes

Are sufficient details of methods and analysis provided to allow replication by others?

Partly

Reviewer Expertise:

veterinary anaesthesia, respiratory physiology

I confirm that I have read this submission and believe that I have an appropriate level of expertise to confirm that it is of an acceptable scientific standard.
